# Does melatonin protect fetal brain during maternal hypothyroidism? An experimental study

**DOI:** 10.12669/pjms.38.5.5536

**Published:** 2022

**Authors:** Mariyah Hidayat

**Affiliations:** Dr. Mariyah Hidayat Professor of Anatomy, University of Health Sciences, Lahore, Pakistan.

**Keywords:** Hypothyroidism, Melatonin, Cytochrome c oxidase enzyme, Mitochondria, Caspases

## Abstract

**Objectives:**

To study the effects of melatonin in preventing neonatal neuronal apoptosis induced by maternal hypothyroidism.

**Methods:**

Twelve healthy female Wistar rats, 12-16 weeks, were divided equally into three groups. Group-A was labelled as control. Group-B was made hypothyroid by giving 15mg/kg of propylthiouracyl (PTU) daily whereas Group-C was given PTU along with melatonin (10mg of melatonin/kg/day) in drinking water. After one week of treatment, the female rats were allowed to mate and conceive. The treatment of all groups continued throughout the period of pregnancy and lactation. After delivery, a total of 30 pups, 10 from each group, were labelled and sacrificed on 22nd day of life. The serum levels of TSH, T3 and T4 of the pups were measured. The brains were extracted from the skull and homogenized for isolation of mitochondria to determine the levels of cytochrome c oxidase and for isolation of RNAs to measure the levels of gene expressions of caspases 3, 9 and 8.

**Results:**

Group-B pups showed a significant increase in serum levels of TSH (21 ± 3.7 mg/dl), and gene expression levels of caspase 3 (0.85±0.02) and 9 (0.69±0.02) where as in Group-C, there was visible reduction in concentration of TSH (15 ± 2.4 mg/dl), caspase 3 (0.50±0.02) and 9 (0.25±0.01) expressions. Increase in cytochrome c oxidase enzyme concentration (3.416 ± 0.001) in Group-B was the result of mitochondrial outer membrane rupture, causing decrease in the number of neurons by accelerating apoptosis. A decrease in its level in Group-C (2.100 ± 0.001) indicated inhibition of apoptosis.

**Conclusion:**

Intake of melatonin during pregnancy and lactation protected the brains of offspring from extensive apoptosis during maternal hypothyroidism.

## INTRODUCTION

Impaired thyroid function can cause mental retardation and other neurological disorders.^1^ From the beginning of development, the nervous system of the embryo is extremely sensitive to thyroid hormones. Abnormalities in thyroid function, especially subclinical hypothyroidism, is highly prevalent in Pakistan^2^,^3^ and one of its major contributing factors is low awareness about the symptoms among people as well as physicians Thyroid Hormone (TH) receptors are found in highest concentration in developing neurons of fetal brain as they are indispensable for the normal growth of both humans and animal brains.^4^ It is a consistent finding of innumerable experimental studies that brain is the main organ which is extremely sensitive to the serum concentration of thyroid hormones.^5^

Melatonin is categorized as an effective antioxidant and anti-apoptotic molecule.^6^,^7^ Although it is primarily synthesized in the pineal gland, but other organs of the body, including the brain, lymphocytes, skin, the gastrointestinal tract, thymus, eye and bone marrow also contribute to its synthesis.^8^ It has been recently reported that melatonin specifically targets mitochondria of all cells, as it is mainly synthesized here.^9^ It is also known that mitochondria is the main organelle involved in the initiation of intrinsic pathway of apoptosis.^10^,^11^ This direct effect of melatonin on mitochondria may be the reason for its anti-apoptotic properties.

Precise mechanism of the apoptosis of the fetal neurons during maternal hypothyroidism is not yet known.^12^ Various animal studies have highlighted the neuroprotective effect of melatonin^13^, but its effect on fetal brain in hypothyroid state has not been explored as yet. Considering the pivotal role of mitochondria in the process of apoptosis and the protective effects of melatonin on mitochondria, the current study investigated the outcome of melatonin administration on the neonatal brains which were exposed to maternal hypothyroidism. Mitochondria associated neuronal apoptosis was mainly focused on. The gene expressions of caspase 3, 8 and 9 were also measured to identify the potential mechanisms of apoptosis induced by the maternal hypothyroidism. These two parameters were chosen for the study as they specifically measure the extent of apoptosis at the tissue level. For ethical reasons, rats are used in the studies as mammalian model.

## METHODS

This experimental study was conducted from January to March, 2020, in the Experimental Research Lab and Department of Anatomy of University of Health Sciences (UHS), Lahore, after purchasing Wistar albino rats from University of Veterinary Animal Sciences, Lahore. Experimental procedures related to animals were performed according to the guidelines laid down by the Ethical Review Committee for medical research at UHS and appropriate measures were taken to reduce the pain of experimental animals. The approved protocol number for this study is UHS/ERC/126-17/20.

Twelve apparently healthy female Wistar rats, 12-16 weeks, in the weight range of 200-250 g, were divided equally into three groups. Group-A served as control and received plain drinking water and chow. Group-B was made hypothyroid by giving 15mg/kg of propylthiouracyl (PTU) daily mixed with chow.^14^ Group-C was given the same medication as Group-B, along with melatonin (10mg of melatonin/kg/day) in drinking water.^15^ After one week of treatment, the female rats were allowed to mate and conceive. The treatment of all groups continued throughout the period of pregnancy and lactation. During this period, the serum levels of TSH, T3 and T4 of the dams were measured weekly. The animals were kept in temperature-controlled room with 22 ± 1ºC and with the light/ dark cycle of 12 h /12 h (light on at 8:00 and off at 20:00 hours). The animals were allowed access to food and water at libitum. Since PTU and melatonin cross placenta easily & secreted in milk, fetuses received these medications from their experimental mothers throughout pregnancy and lactation^16^,^17^ PTU pellets were carefully prepared from the powder and mixed with chow. Melatonin dose was mixed with drinking water.

Pups were sacrificed on 22^nd^ day of life using chloroform soaked in cotton. Blood samples from the pups were immediately collected from the cardiac region for evaluating serum levels of TSH, T3 and T4 using Elisa kit following the instructions of the manufacturer. Neonatal brains were instantly extracted from the skull. Out of the total of 1.3 – 1.5 mg of brain tissue extracted per pup, half was homogenized for isolation of mitochondria and the remaining half was homogenized for RNA extraction to measure the gene expressions of caspases 3, 9 and 8. For isolation of mitochondria, a portion of freshly extracted brain was instantly immersed in ice cold phosphate buffered saline and homogenized in a dounce homogenizer after adding 1ml of isolation buffer to the tissue containing 225 mM sucrose, 75 mM mannitol, 1 mM EGTA and 5 mM Hepes at pH 7.4.^18^ The homogenate was now centrifuged at 1,000xg for 10 minutes at 4°C. The supernatant was collected and further centrifuged at 12,000xg for 15 min at 4°C. Then, 0.5 ml of isolation buffer from the mitochondrial isolation kit and 5μl of protease inhibitor cocktail were added to each pellet and the mixture was centrifuged at 12,000xg for 15 min at 4°C to obtain mitochondria. The pellet now purely contained mitochondria alone, which were used for further experiment. Mitochondrial cytochrome c oxidase has been found to control apoptosis by initiating a cascade of caspases once it is released into the cytosol. For this reason, it was important to measure the concentration of cytochrome c oxidase in the brain tissue of pups from all the three groups to make a comparison of the extent of apoptosis. For recording the enzyme concentration of cytochrome c oxidase inside mitochondria, a cytochrome c oxidase assay kit was purchased and the protocol provided by the manufacturer was followed. Based on its absorbance properties, a spectrophotometer was used and readings recorded at 550 nm wavelength, on the principle that the ruptured membranes will increase the levels of the enzyme, leading to an increase in its absorbance ratio. The spectrophotometric readings obtained were recorded as mean ± SD.

RNA was isolated from the brain tissue by following protocol mentioned in the kit provided. RNA was transcribed to complementary DNA by a thermocycler and Real-time Quantitative Polymerase Chain Reaction (RT-qPCR) was performed in a 11μl of mixture containing 6 μl of SYBR Green PCR Master Mix, 1 μl of sample cDNA, 0.5 μl forward primer, 0.5 μl reverse primer, and 3μl RNAse free water. Data was analyzed to measure the change in the gene expressions of caspases 3, 8 and 9, after normalizing the values with a house keeping gene β-Actin mRNA. One way analysis of variance (ANOVA) was used for normality analysis among groups, followed by Tukey’s post-hoc analysis to obtain the statistical significance of differences of various quantitative changes between the groups. P < 0.05 was considered to be statistically significant and *indicated statistical significance as compared to the rest of the groups. All calculations were done by utilizing computer software SPSS version 20.

## RESULTS

**Fig.1:**
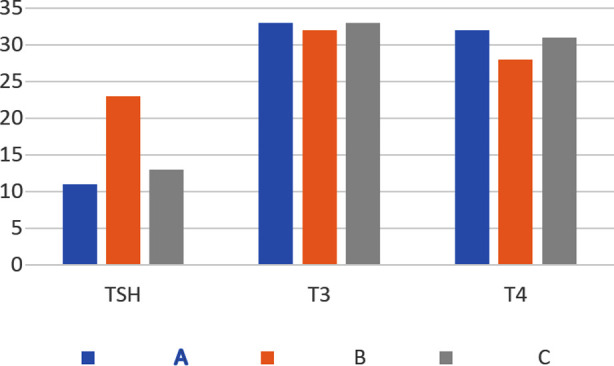
Comparison of mean TSH, T3, T4 in all the groups.

In the control Group-A pups, the serum levels of TSH, T3 and T4 were 10 ± 2.1, 33.7±0.5, 32.1 ± 0.9 mg/dl, respectively. In hypothyroid Group-B pups, the serum levels of TSH, T3 and T4 were 21± 3.7, 32.3 ± 0.4, 27.7 ±1.2 mg/dl, respectively. In melatonin treated Group-C, the serum levels of TSH, T3 and T4 were 15 ± 2.4, 33.3 ± 0.5, 31.3 ±1.2 mg/dl, respectively. The results showed significant increase in serum levels of TSH (P < 0.05) in Group-B compared to A and C (Fig.1). This indicated that melatonin significantly suppressed the rise of TSH induced by PTU (Fig.1). There were no significant alterations observed in serum total T3 levels among groups (Fig.1). In contrast, serum total T4 level was significantly lower in Group-B compared to A. However, in the animals of Group-C co-treated with melatonin, the T4 level was significantly elevated compared to Group-B (p < 0.05) (Fig.1). The mitochondrial levels of cytochrome c oxidase enzyme in groups A, B, C were 1.461± 0.049, 3.416± 0.001, 2.100 ± 0.001, respectively, at the wavelength of 550 nm. Increase in cytochrome c oxidase enzyme concentration (3.416 ± 0.001) in Group-B was the result of mitochondrial outer membrane rupture, causing decrease in the number of neurons by accelerating apoptosis. A decrease in its level in Group-C (2.100 ± 0.001) indicated inhibition of apoptosis.

The data is expressed as mean ± SD (n=10). The numbers in the Y axis are the altered folds of the gene expression. Group-A: control, Group-B: treated with PTU, Group-C: treated with PTU plus melatonin, Group-D: melatonin treated alone.

## DISCUSSION

This study shows a decline in the serum levels of the Thyroxine and increase in TSH levels in the mothers of group B treated with PTU alone and their pups as well. This clearly indicates their hypothyroid state (Table-I). A complex association exists between maternal hypothyroidism and fetal brain activity, as even a slight change in the maternal serum levels of THs can disturb and disrupt functions of mitochondria in fetal brain.^19^

**Table I:** Comparison of mean of TSH, T3, T4, Cytochrome c absorbance ratio among the groups.

Group	TSH(mg/dl)	T3(mg/dl)	T4(mg/dl)	Cytochrome c absorbance ratio at 550nm
A	10 ± 2.1	33.7±0.5	32.1 ± 0.9	1.461± 0.049
B	21* ± 3.7	32.3 ± 0.4	27.7 ±1.2	3.416*± 0.001
C	15 ± 2.4	33.3 ± 0.5	31.3 ±1.2	2.100 ± 0.001
P value	< 0.05*	> 0.05	> 0.05	< 0.05*

Data is expressed as mean ± S.D (n = 10) * P < 0.05 indicates highly significant difference.

Group-A: control, Group-B: treated with PTU, Group-C: treated with PTU plus melatonin.

* Indicates statistical significance as compared to the rest of the groups.

Cytochrome C oxidase enzyme concentration was elevated in Group-B, indicating higher percentage of damage to outer mitochondrial membranes when compared to Group-A and C.

**Table II:** Gene sequences of caspase 3, 8, 9.

Gene	Forward primer (5′- 3′)	Reverse primer (5′- 3′)
CASP 3	GGCCGACTTCCTGTATGCTTAC	GACCCGTCCCTTGAATTTCTC
CASP 8	GATTACGAACGATCAAGCACAGA	ATGGTCACCTCATCCAAAACAGA
CASP 9	TCTGGCAGAGCTCATGATGTCT	GGTGTATGCCATATCTGCATGTCT

This study shows significantly higher levels of the cytochrome c from the brain tissue of the pups born to hypothyroid mothers. This finding is in agreement with the findings of the Sousa N and Miranda A (2018),^19^ who have reported similar result. It has been assumed by the researchers^19^,^20^ that TH deficiency alters mitochondrial morphology of the fetal brain tissue that results in rupture of its outer membrane and outward movement of cytochrome c enzyme from the inter membrane space into the cytoplasm. It is assumed that intrinsic pathway of apoptosis was activated by caspases 3 and 9.^20^ Cytochrome c is released from the inter membrane space of mitochondrion when the serum levels of thyroid hormones are low. To suppress the discharge of this enzyme from the mitochondria is an effective approach to reduce neuronal apoptosis. Melatonin is one of the molecules to have this capacity as treatment with this drug possibly stabilized the mitochondrial membrane with a decline in release of cytochrome c (Table-I). It appears to be a promising drug in reducing the neuronal apoptosis caused due to hypothyroidism.

The effects of THs on brain development and resulting mental deficit due to its deficiency during fetal and neonatal period in humans have been well documented.^21^ Since melatonin is a versatile neuro protector, it is hypothesized that melatonin may provide protective effects on hypothyroid related brain damage in neonates, especially that melatonin has been reported to regulate TH synthesis.^22^ To test this hypothesis, the hypothyroid model was created by treating the pregnant rats with PTU in Group-B and C. This treatment significantly increased the serum levels of TSH and reduced T4 production in Group-B, whereas melatonin administration in Group-C did not allow the serum concentrations of thyroid hormones to rise significantly. This indicates that melatonin antagonized the adverse effects of PTU on thyroid gland in pups. The result was in agreement with the report of de Albuquerque et al. (2020)^23^ in which they found that melatonin directly regulated TH biosynthetic activity of rat thyrocytes and prevented the adverse effects of hypothyroidism on the thyroid gland.

Experiments and investigations have confirmed that cytochrome c is a critical part of mitochondrial electron transport chain and it plays a central role in initiation and regulation of mitochondria associated apoptosis. As an initiator of caspases, cytochrome c triggers series of events leading to cell apoptosis, which attack mitochondria to release more cytochrome c, a vicious cycle comes into play, and eventually, this vicious cycle causes mitochondrial dysfunction with disruption of electron transport chain.^24^ Jia et al. and Du et al. (2021)^25^ have stated that mitochondrial dysfunction is directly related to brain diseases and aging.

Melatonin has widespread protective effects on mitochondria. These include reducing mitochondrial oxidative stress preserving mitochondrial membrane,^26^ upregulating antiapoptotic mitochondrial protein Bcl2, downregulating proapoptotic mitochondrial protein Bax,^27^ and inhibition of caspase 3 activity.^28^ In the current study, melatonin disrupted the vicious cycle of cytochrome c release-mitochondrial damage and effectively reduced the mitochondria associated apoptosis (Table-I). The mitochondrial outer membrane injury in hypothyroid animals might relate to the activation of the intrinsic pathway of apoptosis. In this pathway, the apoptosis is mediated by mitochondria in which the penetrability of the mitochondrial inner membrane is increased. This phenomenon is known as mitochondrial permeability transition with the opening of pores in the mitochondrial inner membrane.^29^

**Fig.2:**
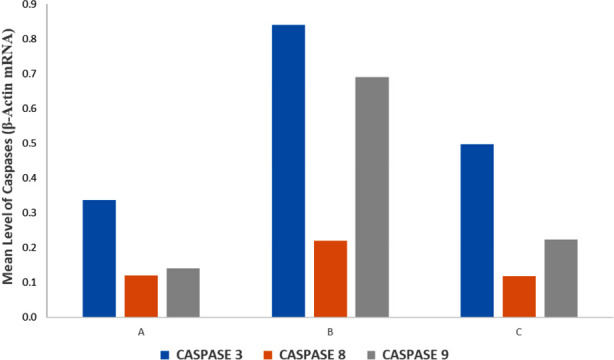
The bar graph of the relative gene expression levels of caspase 3, 8 & 9 under different treatments.

Caspase 9 is able to leak out mainly because of the damage to the outer mitochondrial membrane. This is the initiator caspase which is solely responsible for instigating the intrinsic pathway of apoptosis.^30^ In the present experimental study, the gene expression of Caspase 9 increased in Group-B hypothyroid pups compared to other groups (Table-III, Fig.2). The initiator caspase 9 in turn, activated the executioner caspase 3, which was responsible for apoptosis of the cell (Table-III, Fig.2). Melatonin treated animals of Group-C demonstrated a significant decrease in the gene expressions of caspase 9 and 3, indicating anti-apoptotic effect of this hormone on 22 days old pups (Table-III, Fig.2). On the other hand, caspase 8, which mediates extrinsic apoptotic pathway, presented no notable change in its gene expressions in any of the three subgroups (Table-III, Fig.2), indicating that apoptosis via extrinsic pathway, did not demonstrate any significant change in gene expression, pointing possibly, no role of this pathway in the nervous tissue in hypothyroid state. However, both caspase 3 and 9 are involved in the mitochondrial-mediated apoptosis. It was also confirmed that melatonin mainly protected mitochondria and preserved its membrane potential by inhibition of mitochondrial permeability transition pore, preventing apoptosis.^31^

**Table III:** Altered fold of gene expressions of caspase 3, 8, 9 under different treatments.

Groups	Caspase 3	Caspase 8	Caspase 9
A	0.33 ± 0.04	0.13±0.01	0±0.01
B	0.85*±0.02	0.23±0.02	0.69*±0.02
C	0.50±0.02	0.12±0.01	0.25±0.01

Group-A: control, Group-B: treated with PTU, Group-C: treated with PTU plus melatonin.

* Indicates statistical significance vs the rest of the groups (* P < 0.05).

### Limitation of this study:

For ethical reasons, this study could not be performed on humans, which is the main limitation of this study.

## CONCLUSION

The results of this study showed that maternal hypothyroidism caused neuronal apoptosis of the neonates via the mitochondrial pathway and melatonin shows a potential against protecting mitochondrial injury. Since there are limited remedies to effectively treat hypothyroidism related neonatal brain damage, the results may lead to the development of potential treatment strategies related to the use of melatonin in the hypothyroid mother.
